# Influence of Chondroitin Sulfate Concentration on the Physicochemical Properties of Acetaminophen-Releasing Cellulose/PVOH Membranes

**DOI:** 10.1021/acsomega.6c01092

**Published:** 2026-04-16

**Authors:** Juliano Brisola, Paula Paulino Silva, Francisnara Tonholi, Jéssica Bassetto Carra, Rúbia Casagrande, Paulo Rodrigo Stival Bittencourt, Gizilene Maria de Carvalho

**Affiliations:** † Department of Chemistry, State University of Londrina, Londrina 86057-970, Brazil; ‡ Department of Pharmaceutical Sciences, State University of Londrina, Londrina 86057-970, Brazil; § Department of Chemistry, Federal University of Technology—Paraná (UTFPR), Medianeira Campus, Medianeira 85722-332, Paraná, Brazil

## Abstract

The integrity of the skin barrier is essential for protecting the body from external threats. However, mechanical trauma, thermal injuries, and chronic wounds can impair this barrier and increase susceptibility to secondary infections. Wound dressings capable of in situ drug release and skin regeneration have emerged as effective strategies to promote accelerated healing. Cellulose/poly­(vinyl alcohol) (Cel/PVOH) and cellulose/chondroitin sulfate (Cel/CS) membranes are particularly promising for this purpose. However, it is essential to understand how increasing the chondroitin sulfate content affects the physical and chemical properties of these membranes. In this study, Cel/PVOH membranes incorporated with acetaminophen were developed, with varying amounts of chondroitin sulfate. Structural characterization by FT-IR and XRD revealed that higher CS content reduced crystallinity, thermal stability, and water vapor permeability. SEM analysis showed increased surface porosity associated with CS incorporation. Membranes with higher CS levels exhibited enhanced fluid handling capacity (FHC), ranging from 3.48 to 5.15 g·10 cm^–2^·24 h^–1^. Drug release studies demonstrated a diffusion-controlled kinetic profile for acetaminophen. This work presents a simple method for fabricating membranes with desirable physicochemical and structural properties, highlighting their potential for use as bioactive dressings and transdermal drug delivery systems.

## Graphical abstract

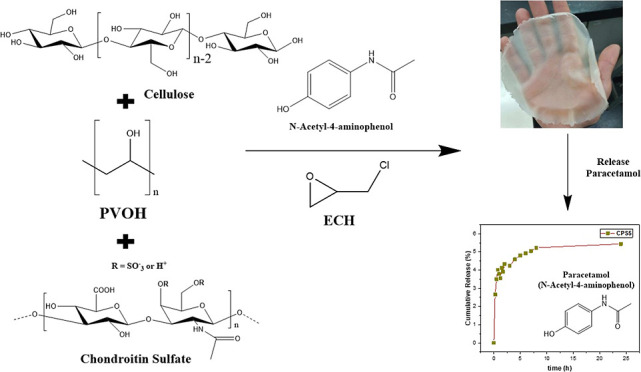

## 1 Introduction

Skin is the largest organ of the human body and serves as the primary physical barrier against pathogens and harmful agents.^1^ Damage to this protective layer, caused by mechanical trauma, thermal injuries, or chronic wounds, compromises its structural integrity and is frequently associated with a high risk of secondary infections.^2^

With the advancement of medicine, wound healing procedures have become increasingly diversified.^3^ The use of wound dressings capable of in situ drug release directly at the wound site stands out due to their ease of application and ability to reduce healing time.^4^

Natural polymers have gained prominence in the production of these membranes due to their hydrophilicity, mechanical strength, and compatibility with living tissues.^5^ Although poly­(vinyl alcohol) (PVOH) possesses favorable properties, its application is limited by low mechanical resistance.^6^ In this context, cellulose (Cel) and its derivatives have been employed in the production of blends and composites with PVOH, as they impart enhanced mechanical strength,^7,8^ in addition to enabling porosity tuning to modulate properties such as swelling behavior and drug release (poly­(vinyl alcohol)-cellulose nanocrystal hydrogel containing anti-inflammatory agent).^9,10^

Chondroitin sulfate (CS), a glycosaminoglycan abundant in connective tissues, further expands the functionality of biopolymer blend. Composed of alternating *N*-acetyl galactosamine and glucuronic acid units linked by β(1 → 3) and β(1 → 4) bonds, CS enhances cell adhesion^11^. Previous studies have demonstrated its utility in drug delivery systems and scaffolds, including cellulose-CS hydrogels for controlled drug release^12,13^ and thermosensitive CS-based.^14^ Additionally, CS has been combined with chitosan and gelatin to create electrospun bioadhesive membranes.^15,16^

Although the potential of Cel/PVOH and Cel/CS membranes has been investigated to some degree, the influence of varying CS concentrations within Cel/PVOH systems remains insufficiently explored, particularly regarding their water absorption kinetics and drug release profiles, which are critical parameters for dressing design. This study addresses this gap by developing and characterizing cellulose/PVOH membranes using acetaminophen as a model drug, and by investigating the effect of increasing CS. The structural properties of the membranes are evaluated, and drug release kinetics are analyzed using established mathematical models (Korsmeyer–Peppas, Peppas–Sahlin, and Schott) to elucidate the underlying transport mechanisms.

## 2 Experimental Section

### 2.1 Materials

Microcrystalline cellulose (MCC) GP = 350, CAS:9004-34-6, sodium hydroxide (99%), CAS: 1310-73-2, sulfuric acid (98%) CAS: 7664-93-9, urea (≥99%, CAS: 57-13-6) and ECH (Epichlorohydrin, CAS 106-89-8) were purchased from Synth, Brazil. Poly­(vinyl alcohol) (PVOH) molar mass 540,000 g·mol^–1^ CAS 9002-89-5, degree of hydrolysis 88.14% was purchased from Selvol 203 (Sekisui Chemical, Japan). Chondroitin Sulfate (CS) was donated by the company Adeste, Jaguapitã-PR. Cellulose nitrate membrane, 0.45 μm pores, were purchased from Sartorius Stedim, CAS: 9004-70-0, Biotech, Germany.

### 2.2 Membrane Production and Acetaminophen Incorporation

The cellulose solution was prepared according to the method described by Cai and Zhang,^17^ with modifications. Specifically, 3 g of microcrystalline cellulose were added to 100 mL of a NaOH/Urea/H_2_O solution (7%/12%/81% by mass) at room temperature under continuous stirring for 30 min. The system was then stored at −5 °C for 20 h. Subsequently, the solution was thawed under stirring for 1 h. The PVOH solution was prepared by dissolving 3 g of the polymer in 100 mL of distilled water at 60 °C, followed by continuous stirring for 1 h. Similarly, the CS solution was prepared by dissolving 3 g of the polymer in 100 mL of distilled water at 25 °C, with stirring for 2 h. Blend membranes were obtained by mixing the polymer solutions in different proportions under magnetic stirring at 25 °C for 1 h (Table 1). ECH was incorporated into all membrane formulations at a concentration of 7% relative to the total solution volume. ECH was selected as a cross-linking agent due to its well-established reactivity toward hydroxyl groups of polysaccharides and poly­(vinyl alcohol), enabling the formation of stable ether linkages and chemically cross-linked polymer networks.^18^ After ECH addition, the mixtures were maintained at 60 °C under stirring for 1 h, poured into Petri dishes, and subsequently dried in an oven at 50 °C for 24 h. The obtained membranes were then subjected to a regeneration process using 5% (v/v) H_2_SO_4_ for 20 min, repeated three times. Following regeneration, the membranes were thoroughly washed with distilled water until the pH reached approximately 7 and removed any unreacted reagents.^19^ Finally, the membranes were dried in an oven at 50 °C for an additional 24 h.

**Table 1:** Mixtures Composition in the Filmogenic Solutions Formulated with Cellulose, Poly­(vinyl alcohol) (PVOH), Chondroithin Sulfate (3% w/v) and Acetaminophen

solutions samples	celulose (mL)	PVOH (mL)	CS (mL)	acetaminophen (g)
Cel	93	0	0	0
PVOH	0	93	0	0
CP	46.5	46.5	0	0
CPS1	41.5	41.5	10	0
CPS2	36.5	36.5	20	0
CPS3	31.5	31.5	30	0
CPS5	21.5	21.5	50	0
CPP	46.5	46.5	0	1
CPS1P	41.5	41.5	10	1
CPS2P	36.5	36.5	20	1
CPS3P	31.5	31.5	30	1
CPS5P	21.5	21.5	50	1

To prepare the membranes containing acetaminophen, 1 g of the drug was incorporated into the polymer solutions, maintaining the same compositions as described in Table 1, prior to the addition of ECH. The mixtures were stirred continuously until the complete dissolution of the acetaminophen was achieved. Following this step, the procedure was carried out as described in the previous section.

### 2.3 Characterizations

#### 2.3.1 FTIR–ATR, WAXD, TGA and DSC

Fourier transform infrared (FTIR–ATR) spectra were recorded using a Bruker Vertex 70 spectrophotometer equipped with a Platinum ATR reflectance accessory. The measurements were conducted with a spectral resolution of 4 cm^–1^ and 16 scans. The spectra were normalized within the range of 0 to 1.

Wide-angle X-ray diffraction (WAXD) measurements were performed using a panalytical X’Pert PRO MPD diffractometer operating in Bragg–Brentano geometry with CuKα radiation. The 2 θ scan range was set from 5° to 30°, with an angular step of 0.05°. The applied voltage and current were 40 kV and 30 mA, respectively, and the counting time per point was 2.0 s. X-ray diffraction analysis is commonly employed to evaluate the degree of crystallinity in semicrystalline polymers. The relative crystalline (% CI) of cellulose and the membranes were determined by deconvoluting the obtained WAXD patterns to separate the contributions of the crystalline (*A*_c_) and amorphous (*A*_am_) regions, following the methodology proposed by Park^20^ (eq 1). The crystalline contribution was modeled using a pseudo-Voigt function, which represents a mixture of Gaussian and Lorentzian distributions, while the amorphous contribution was fitted using a Gaussian function.

1 $$\%\;CI=\frac{A_{c}}{A_{c}+{\;A}_{am}}\times 100$$

Thermogravimetric analysis (TGA) was conducted using a Shimadzu TGA 50 (Japan) under a nitrogen atmosphere with a flow rate of 50 mL min^–1^. Approximately 10 mg of each sample was heated from 30 to 800 °C at a heating rate of 20 °C min^–1^.

Differential scanning calorimetry (DSC) was performed using a Shimadzu DSC 60 (Japan) calorimeter. Approximately 3.0 mg of each sample was placed in platinum crucibles and heated from 30 to 300 °C at a heating rate of 10 °C·min^–1^ under a nitrogen atmosphere with a flow rate of 50 mL min^–1^.

#### 2.3.2 Scanning Electron Microscopy (SEM) Analysis

SEM analyses were performed using a FEI Quanta 200 microscope (Oregon, USA) to examine the surface morphology and cryofracture characteristics of the membranes. The dried samples were affixed to bronze stubs using double-sided adhesive tape and subsequently coated with a thin gold layer (40–50 nm) to enhance conductivity. Imaging was conducted at an accelerating voltage of 25 kV.

### 2.4 Physicochemical Analyses

#### 2.4.1 Moisture Content (% TU)

Dried membrane samples were cut into 2.0 × 2.0 cm sections and immersed in distilled water at 25 °C for 1 h (*W*_s_). After immersion, the samples were removed and dried in an oven at 100 °C for 24 h, after which their dry mass (*W*_d_) was recorded. The moisture content was determined based on the mass difference between the wet and dry samples, using eq 2.

2 $$\%\;TU=\frac{W_{s}}{W_{d}}\times 100$$

#### 2.4.2 Swelling Analysis of Membranes

Membrane samples measuring 2.0 × 2.0 cm were cut, dried in an oven at 40 °C for 72 h, and subsequently weighed to determine their initial dry weight (*W*_D_). Each sample was then immersed in a vial containing 50 mL of water at room temperature. At predetermined time intervals, the samples were removed from the water, gently blotted to remove surface moisture, and weighed again (*W*_S_). Measurements were taken periodically until swelling equilibrium was reached. The degree of swelling (% SW) was calculated using eq 3.

3 $$\%\;SW=\frac{W_{S}-W_{D}}{{\left(W\right)}_{D}}\times 100$$

#### 2.4.3 Determination of Water Loss (WL)

The water loss content was performed in triplicate according to Tan.^21^ The 2.0 ×2.0 cm samples were hydrated at room temperature for 1 h. After this time, the samples were transferred to previously weighed glass plates and stored in an oven at 50 °C. The samples were removed from the oven and their masses were determined at the following times: 0 min, 15 min, 30 min, 1, 2, 3, 4, 5, 6, 7, 8, and 24 h. The water loss was calculated using eq 4.

4 $$\%\;WL=\frac{M_{t}-M_{i}}{M_{e}-M_{i}}\times 100$$

where *M*_e_ is the mass of the wet sample at equilibrium; *M*_*t*_ is the mass of the sample at time (*t*); *M*_i_ is the mass of the dry sample.

#### 2.4.4 Determination of Water Vapor Permeability (WVP)

Thickness was determined using a manual micrometer with ±0.1 μm accuracy (Mitutoyo, Brazil), and calculated as the average of 10 measurements taken at random positions on the membranes. WVP was performed according to a modified ASTM-E9695 method.^22^ Film samples were sealed over a 30 mm circular opening of a permeation cell containing calcium chloride (0% RH inside the cell). The set was placed inside a desiccator containing saturated sodium chloride solution (75% RH outside the cell) to create a 75% RH gradient across the film. The water vapor permeability was calculated according to eq 5.

5 $$WVP=\frac{g}{t}\times \frac{e}{A\times \Delta P}$$

where *g*/*t* is the slope of the line obtained by linear regression of the mass gain (g) vs conditioning time (s) graph. *A* is the permeation area of each specimen. e is the thickness of each sample. Δ*P* is the pressure difference between the environment containing calcium chloride and the environment containing saturated NaCl, with a value of 2375.4 Pa.

#### 2.4.5 Fluid Handling Capacity (FHC)

The evaluation of the FHC was performed using a Simulated exudate fluid (SEF), following the guidelines outlined in British Standard BSEN 13726-1:2002,^23^ with slight methodological modifications. The SEF was prepared as a saline solution composed of 0.142 mol·L^–1^ sodium chloride (NaCl) and 0.0025 mol·L^–1^ calcium chloride (CaCl_2_), which reflect the average ionic concentrations typically found in wound exudate. The test membranes were mounted in modified permeability test cells, each consisting of two compartments: one allocated for SEF application and the other containing an orifice (7.0 cm^2^) to allow fluid permeation through the membrane. A volume of 7.0 mL of SEF was introduced into the upper compartment of each cell. The membranes were secured over the orifice using stainless steel clips, and a layer of fabric with the same diameter as the membrane was placed on top to enhance mechanical stability. The entire assembly (membrane, fluid, and support fabric) was weighed and then inverted and incubated in a desiccator maintained at 37 ± 1 °C and relative humidity below 20% for 24 h. Following the incubation period, the assemblies were equilibrated at ambient temperature (approximately 25 °C) for 30 min, and their final mass was recorded. Subsequently, the membranes were removed, gently drained of excess fluid, and weighed.

The moisture vapor transmission rate (MVTR), the absorbed fluid mass (ABS), and the total fluid handling capacity (FHC) were calculated based on gravimetric data using the eqs 6 to 8.

6 $$MVTR=\frac{W_{2}-W_{3}}{t\times A}$$

7 $$ABS=\frac{W_{4}-W_{1}}{t\times A}$$

8 FHC=MVTR+ABS

where: TPVU = moisture vapor permeability; CA = absorptive capacity; CMF = fluid movement capacity; *W*_2_ = initial mass of the system; *W*_3_ = final mass of the system after 24 h; *W*_1_ = initial mass of the membrane; *W*_4_ = final mass of the membrane after 24 h; *t* = storage time (24 h); *A* = membrane area (cm^2^); the results were statistically analyzed by the Tukey test and analysis of variance, using the R statistica software.

### 2.5 Franz Diffusion Cell Assays for Acetaminophen Release

The release of acetaminophen was determined following the methodology described by Ribeiro^24^ with modifications. The receptor compartment, with a volumetric capacity of 10 mL, was filled with phosphate-buffered saline solution (PBS, pH 7.4) and maintained under constant agitation. Synthetic cellulose nitrate membranes (Sartorius Stedim Biotech GmbH, pore diameter 0.45 μm) were positioned in the cell between the donor and receptor compartments. The polymer blends, incorporated with acetaminophen, were cut into disc with a diameter of 1.5 cm and an area of 1.76 cm^2^, and were placed in the donor/receptor system. The system was kept at a temperature of 32 °C with continuous agitation at 300 rpm.

Aliquots (3 mL) were collected at time intervals ranging from 0 to 24 h, and the permeation of acetaminophen (*N*-acetyl-4-aminophenol) was determined using a Shimadzu UV/vis spectrophotometer, model UV-2600, with a detection wavelength of 243 nm. PBS solution (pH 7.4) served as the blank in this experiment. To calculate the amount of permeated of acetaminophen, the analytical curve was constructed in the concentration range of 0.001 to 0.02 mg mL^–1^, with the equation: Abs = 65.000 ± 1*x* + 0.020 ± 0.01 (as illustrated in Figure S1 of the Supporting Information).

For each aliquot withdrawn, 3.0 mL of the PBS solution was returned to the system to maintain a constant volume. The amount of acetaminophen released was calculated by correcting the dissolution value at each time point, accounting for the amount of permeated acetaminophen (*C*_s_) over time (*t*). After the first sampling, the concentration values in each dilution were determined using eq 9, in accordance with Aronson.^25^

9 $$Q_{i}=C_{i}\times V_{i}+\sum \left(C_{s}\times V_{S}\right)$$

where *Q*_i_ is the cumulative amount of acetaminophen permeated. *C*_i_ is the concentration in the current sample. *V*_i_ is the volume of the diffusion cell receptor solution, *C*_s_ is the concentration of the sample removed, and *V*_s_ is the volume of the sample removed.

## 3 Results and Discussion

### 3.1 Membranes Production

The synthesis of Cel/PVOH/CS membranes cross-linked with ECH, with and without the presence of acetaminophen, resulted in flexible, translucent, and homogeneous membranes, as shown in Figure 1a. Additionally, the membranes did not exhibit any cracks on their surface, which may suggest that the inclusion of PVOH contributes to the increased flexibility of the membranes. This effect was also observed by Xie,^26^ who synthesized cellulose blends with PVOH cross-linked with ECH for packaging applications, and reported that the incorporation of PVOH enhanced the mechanical resistance of the membranes.

**Figure 1:**
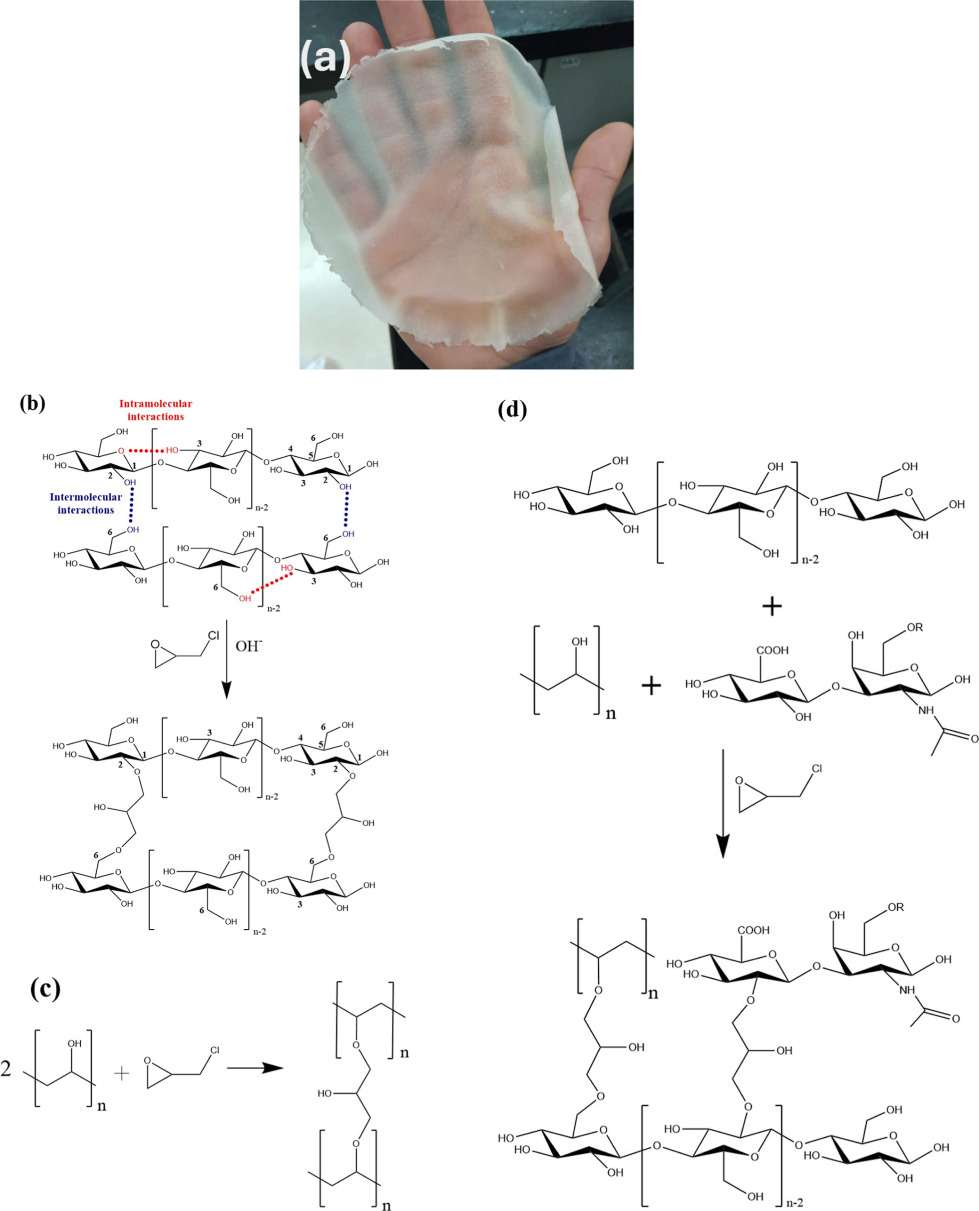
(a) Representative image of the cellulose-based membrane containing 50% chondroitin sulfate (CPS5), in the absence of acetaminophen. (b) Schematic representation of the cross-linking reaction between cellulose and epichlorohydrin. (c) Schematic representation of the cross-linking reaction between poly­(vinyl alcohol) (PVOH) and epichlorohydrin. (d) Detailed mechanism of the cross-linking reaction involving cellulose, PVOH and CS.

Epichlorohydrin, which is reactive with hydroxyl groups, is widely used for cross-linking cellulose and PVOH.^27–30^ The reaction occurs through the opening of the epoxy ring of ECH in a basic medium, causing cross-linking between two cellulose chains via the OH groups at C6 and C2 (Figure 1b).^31^ Similarly, ECH also cross-links with PVOH (Figure 2c). The epoxy group reacts with the hydroxyl group of PVOH,^32^ as shown in Figure 1c, and cross-linking may also occur between cellulose and PVOH chains (Figure 1d).

**Figure 2:**
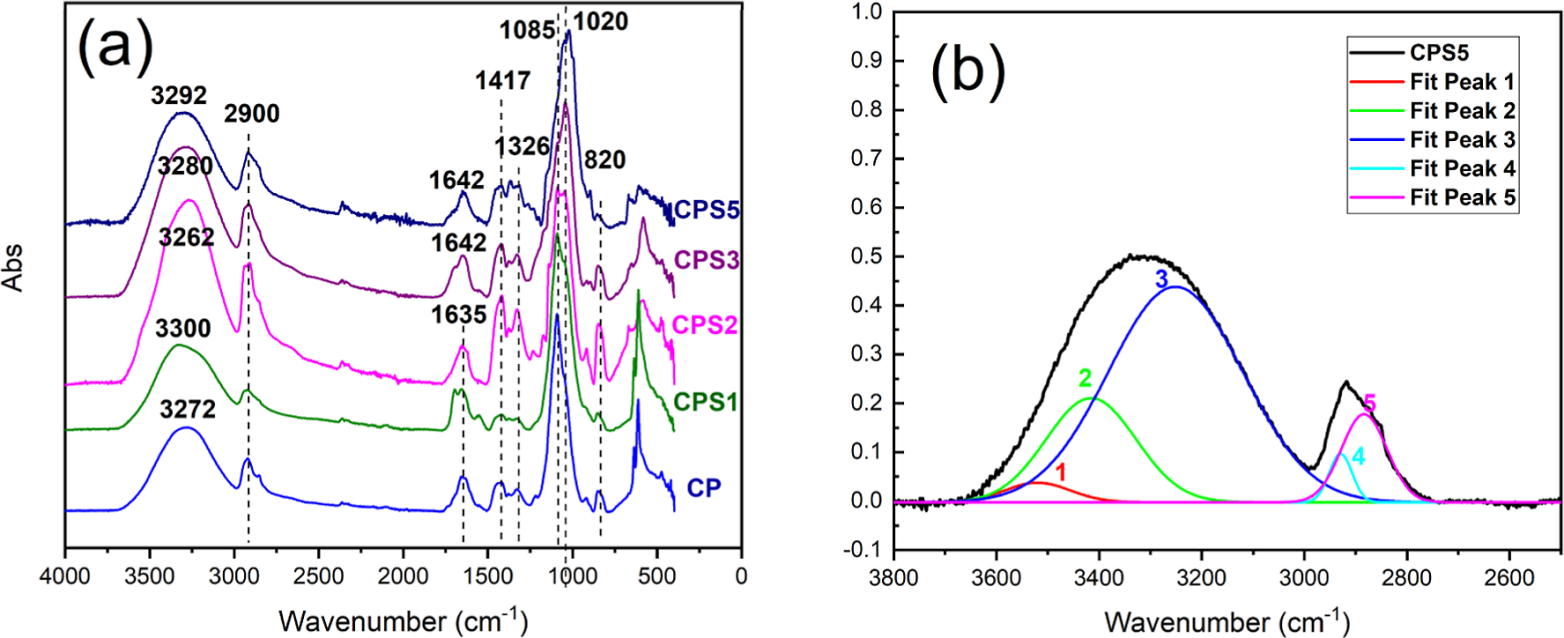
ATR-FTIR spectral analysis of the materials under study. (a) ATR-FTIR spectra of cellulose/PVOH membranes containing different concentrations of chondroitin sulfate. (b) Deconvolution of the region from 2600 to 3600 cm^–1^ of sample CPS5.

### 3.2 FTIR–ATR

The main FTIR absorption bands identified in the spectra are summarized in Table 2, which provides the assignments of the characteristic vibrational modes observed for cellulose, PVOH, chondroitin sulfate, and the produced membranes.

**Table 2:** Main Wavenumbers Identified in the FT-IR Spectra of Cel, CS, PVOH, CPS1, CPS2, CPS3, and CPS5 Samples

wavenumber (cm^–1^)	assignment	structural origin
3260–3300	O–H stretching vibration of hydrogen-bonded hydroxyl groups	cellulose and PVOH hydroxyl groups involved in intra- and intermolecular hydrogen bonding
∼3319	N–H stretching vibration	amide groups from chondroitin sulfate
2890–2900	C–H stretching vibration of –CH_2_ and –CH_3_ groups	polysaccharide backbone and PVOH chains
1635–1642	H–O–H bending vibration of absorbed water	physically adsorbed water in hydrophilic polymer network
∼1235	S=O stretching vibration	sulfate ester groups (−OSO_3_^–^) from chondroitin sulfate
1085	C–O–C asymmetric stretching vibration	glycosidic bonds of cellulose and ether linkages formed during epichlorohydrin cross-linking
∼1020	C–O stretching vibration of polysaccharide ring	cellulose and chondroitin sulfate backbone

Figure S2a,b (material supplementary) shows the normalized spectra of the raw materials used in membrane production. The characteristic bands of cellulose, PVOH, and chondroitin sulfate are consistent with those reported in the literature, including the broad O–H stretching region associated with hydrogen-bonded hydroxyl groups, the C–H stretching vibrations of the polymer backbone, and the characteristic sulfate group vibrations of chondroitin sulfate.^13,32,33^

The spectra of the membranes (Figure 2a**)** exhibit similar band patterns to those observed for the neat components, indicating that the fundamental structural units of the polymers are preserved after membrane formation. A broad band between approximately 3272 and 3300 cm^–1^ is associated with hydroxyl stretching in the polysaccharide network, while the band around 1635–1642 cm^–1^ corresponds to vibrations of absorbed water in the hydrophilic matrix.^34,35^ Additional bands assigned to cellulose backbone vibrations are observed in the 1085–1020 cm^–1^ region.^36^ The band in the region around 820 cm^–1^ corresponds to the stretching vibration of the C–O–S group in CS.^37^

Indirect evidence of the cross-linking reaction with epichlorohydrin is observed in the 950–1400 cm^–1^ region (Figure 2b), where the increase in the C–O–C stretching band intensity suggests the formation of ether linkages. The band near 1326 cm^–1^, which is commonly attributed to CH_2_ wagging and O–H bending vibrations in cellulose, may also be influenced by the formation of ether linkages during epichlorohydrin-mediated cross-linking.^30,38,39^

Because FTIR spectra of cellulose-based materials often present overlapping bands, additional insight into structural changes was obtained through spectral deconvolution of the 3600–3000 cm^–1^ region, which allows the identification of hydrogen-bonding interactions within the cellulose network. This approach enables the evaluation of intra- and intermolecular hydrogen bonds associated with the O(3)­H···O(5), O(3)­H···O(6), and O(2)­H···O(6) interactions characteristic of cellulose II^38,42^ ]. The influence of chondroitin sulfate incorporation, PVOH addition, and epichlorohydrin cross-linking on the hydrogen-bonding network was evaluated through the positions of the deconvoluted bands and the calculated hydrogen bond energy (EH), as presented in Table 3.

**Table 3:** Position and Energy of Hydrogen Bonds in Cellulose and Produced Membranes

	peak 1		peak 2		peak 3	
samples	wavenumber (cm^–1^)	energy (kJ)	wavenumber (cm^–1^)	energy (kJ)	wavenumber (cm^–1^)	energy (kJ)
MCC	3448	11	3298	22	3133	34
Cel	3388	15	3336	19	3196	29
CP	3362	18	3203	28	3099	36
CPS1	3543	4	3378	16	3179	30
CPS2	3527	5	3409	14	3261	25
CPS3	3543	4	3403	13	3238	26
CPS5	3526	6	3414	13	3251	25

A useful IR signature band for the formation of cross-linked is the C–O band at ∼950–1400 cm^–1^, where new peaks are evident due to the formation of new C–O–C bonds.^38^ The increase in intensity of this band for the blends (Figure S2b) when compared to that of MCC provide additional support for the cross-linking reaction in Figure 1d. The band at 1326 cm^–1^ in the cross-linked membranes demonstrated the successful ether-based cross-linking of modified cellulose with ECH.^30,39^

The similarity observed in the IR spectra of pristine cellulose and the cross-linked blends suggests that the fundamental structural units are maintained. However, the structural analysis of the cellulose, PVOH, and CS blends, particularly in relation to their composition and the cross-linking reaction with ECH, presents a considerable challenge. A potential approach to investigate these structural modifications is through deconvolution of the broad O–H stretching region (3600–3000 cm^–1^).

In this study, the dissolution of cellulose using a NaOH/urea/water solvent system favored the formation of cellulose II, which exhibits an antiparallel arrangement of polymer chains. This configuration results in unique distances and orientations between functional groups, giving rise to a distinct hydrogen-bonding network.^40–43^

The torsion angle O5–C5–C6–O6 at the C6 primary alcohol group is defined as ω (Figure 3a). The dihedral angle ω can adopt three stable conformations in the various polymorphs of cellulose: gauche–trans (gt, ω = 60°), trans–gauche (tg, ω = 180°), and gauche–gauche (gg, ω = 300°). For cellulose II, the preferred conformation is tg, which favors intramolecular hydrogen bonding between adjacent glucopyranose units, specifically between O(3)­H···O(5), with O(3)­H···O(6) as a minor component Figure 3b.^40,44^

**Figure 3:**
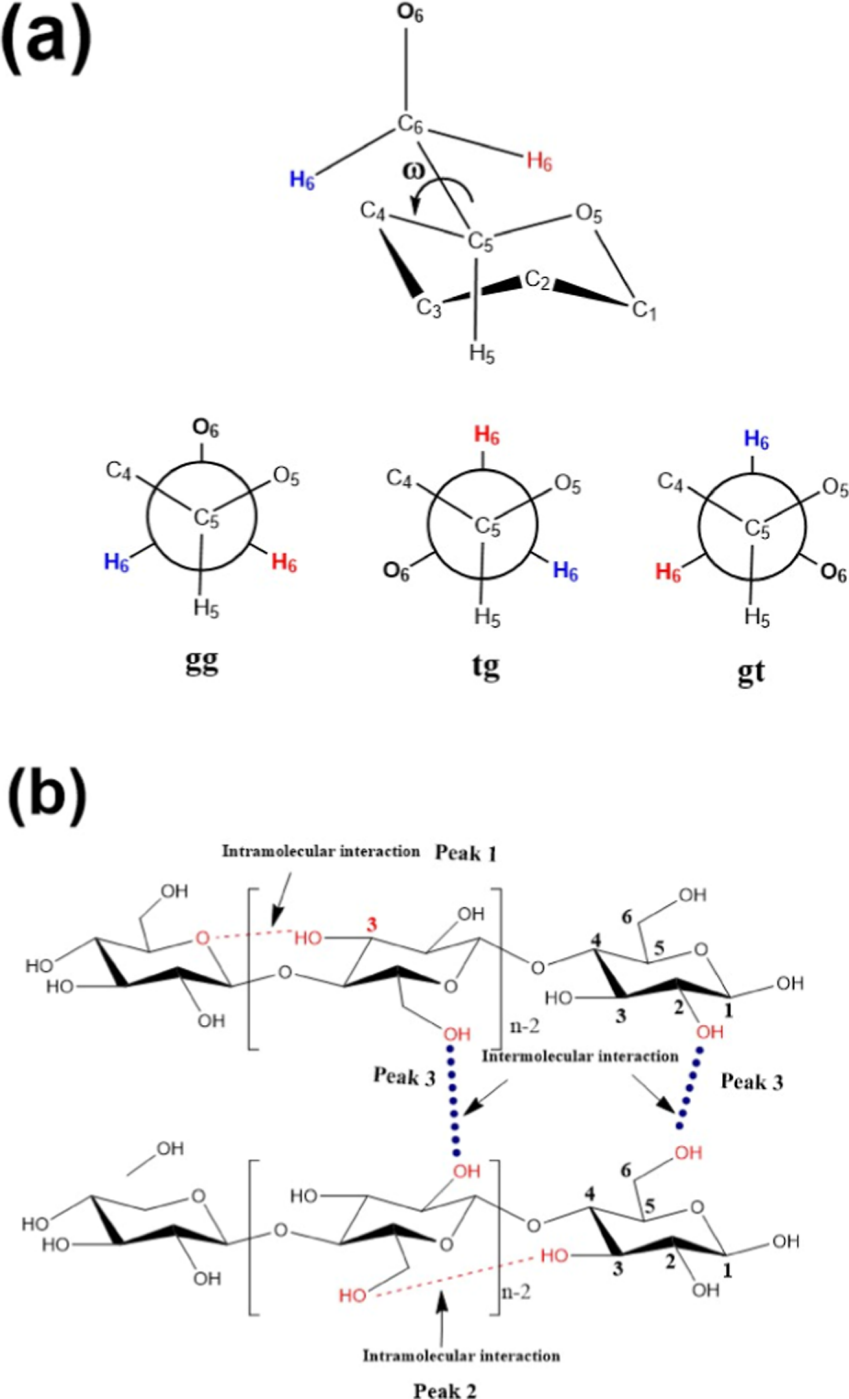
(a) Positioning of the torsion angle of cellulose II and rotational positions of the hydroxymethyl group.^46^ (b) Structure of cellulose II, showing hydrogen bonding network.

To resolve and assign specific hydrogen bonding interactions in cellulose II using FTIR spectroscopy, spectral deconvolution deconvolutions of the 3600–3000 cm^–1^ region were performed for all membranes, using Gaussian function as shown in the Supporting Information, Figure S3a to g. Absorbance of the band obtained from a local baseline between adjacent valleys was automatically calculated at the maximum absorbance found by OriginPro 2018 software. The three signals obtained in the deconvolution can be attributed to different OH bonds present in the cellulose structure.^45^ Intramolecular bonds O(3)­H----O(5) (PEAK 1) and O(3)­H----O(6) (PEAK 2) and intermolecular bonds O(2)­H----O6 (PEAK 3) (Figure 3A).^36,46–49^

The influence of chondroitin sulfate (CS) and poly­(vinyl alcohol) (PVOH) incorporation, as well as the cross-linking reaction with epichlorohydrin, on the strength of inter- and intramolecular hydrogen bonds within cellulose chains (Cel–Cel), was evaluated based on the peak positions in the FTIR spectra. The corresponding hydrogen bond energy (*E*_H_), determined through spectral deconvolution and calculated according to eq 10, is summarized in Table 2.^50,51^

10 $$E_{H}=\frac{1}{k}\frac{\nu_{0}-\nu }{\nu_{0}}\;\left[kJ\right]$$

where: ν_0_ represents the standard frequency of free hydroxyl (–OH) groups, with a reference value of 3600 cm^–1^; ν corresponds to the vibrational frequency of hydrogen-bonded –OH groups (in cm^–1^); *k* is the bond force constant, with its inverse defined as 1/*k* = 262.5 kJ.

Table 3 presents the wavenumbers and corresponding hydrogen bond energies of the membranes composed of cellulose (Cel), cellulose/PVOH (CP), and cellulose/PVOH/chondroitin sulfate (CPS1, CPS2, CPS3, CPS5), all cross-linked with ECH. Peaks 1 and 2 are attributed to intramolecular hydrogen bonds, whereas peak 3 corresponds to intermolecular interactions.

A shift to higher wavenumbers for peak 3 was observed when comparing microcrystalline cellulose (MCC) to Cel and CP, suggesting a reduction in intermolecular hydrogen bond strength.^52^ This is attributed to the action of ECH, which introduces cross-links that act as spacer groups between polymer chains, thereby disrupting intermolecular associations. Conversely, a shift to lower wavenumbers (from 3196 to 3099 cm^–1^) for peak 3 was identified when comparing Cel and CP, indicating enhanced intermolecular hydrogen bonding between O(2)–H···O(6) groups within the cellulose–PVOH matrix. This suggests improved compatibility and interaction between the biopolymers in the blend. The incorporation of CS led to a progressive shift of peak 3 toward higher wavenumbers in CPS1–CPS5 relative to CP, signifying a gradual weakening of the O(2)–H···O(6) intermolecular hydrogen bonds as CS concentration increased. This trend is consistent with the disruptive effect of CS on the hydrogen bonding network, likely due to its highly anionic nature and steric hindrance. These observations are supported by data shown in Supporting Information, Figure S3a to g, as well as the quantitative data in Table 3.

Hydrogen bond energy analysis further supports these findings. A reduction in intermolecular hydrogen bond energy was observed from MCC to Cel, reinforcing the hypothesis of bond weakening due to cross-linking. In contrast, the CP membrane exhibited an increase in hydrogen bond energy, consistent with the formation of new interactions between cellulose and PVOH. However, the addition of CS led to a subsequent decrease in hydrogen bonding energy across the CPS membranes, aligning with the spectral shifts discussed above.

Similar trends were noted for the intramolecular hydrogen bonds represented by peaks 1 and 2, indicating that both intra-and intermolecular hydrogen bonding interactions are significantly influenced by the presence and concentration of CS.

The results presented in Table 3 indicate a decrease in the hydrogen bond energy associated with peak 3, which corresponds to the intramolecular O(3)–H···O(5) interactions. Specifically, the energy decreased from 34 kJ in the MCC sample to 29 kJ in the Cel membrane. This reduction is attributed to the lower number of available hydroxyl groups for hydrogen bonding, resulting from the cross-linking reaction with epichlorohydrin. Furthermore, a continued reduction in hydrogen bond energy for peak 3 was observed in the CPS1, CPS2, CPS3, and CPS5 membranes. This trend suggests that both the increasing concentration of chondroitin sulfate (CS) and the cross-linking process contribute synergistically to the weakening of intramolecular hydrogen bonding within the cellulose chains.

### 3.3 XRD Analysis

The XRD diffractograms of MCC, PVOH, CS and the studied blends are shown in Figure 4a. MCC showed characteristic peaks of type I cellulose at 2θ = 15°, 22°, and 34°, corresponding to the 101, 200, and 040 planes, respectively.^53^

**Figure 4:**
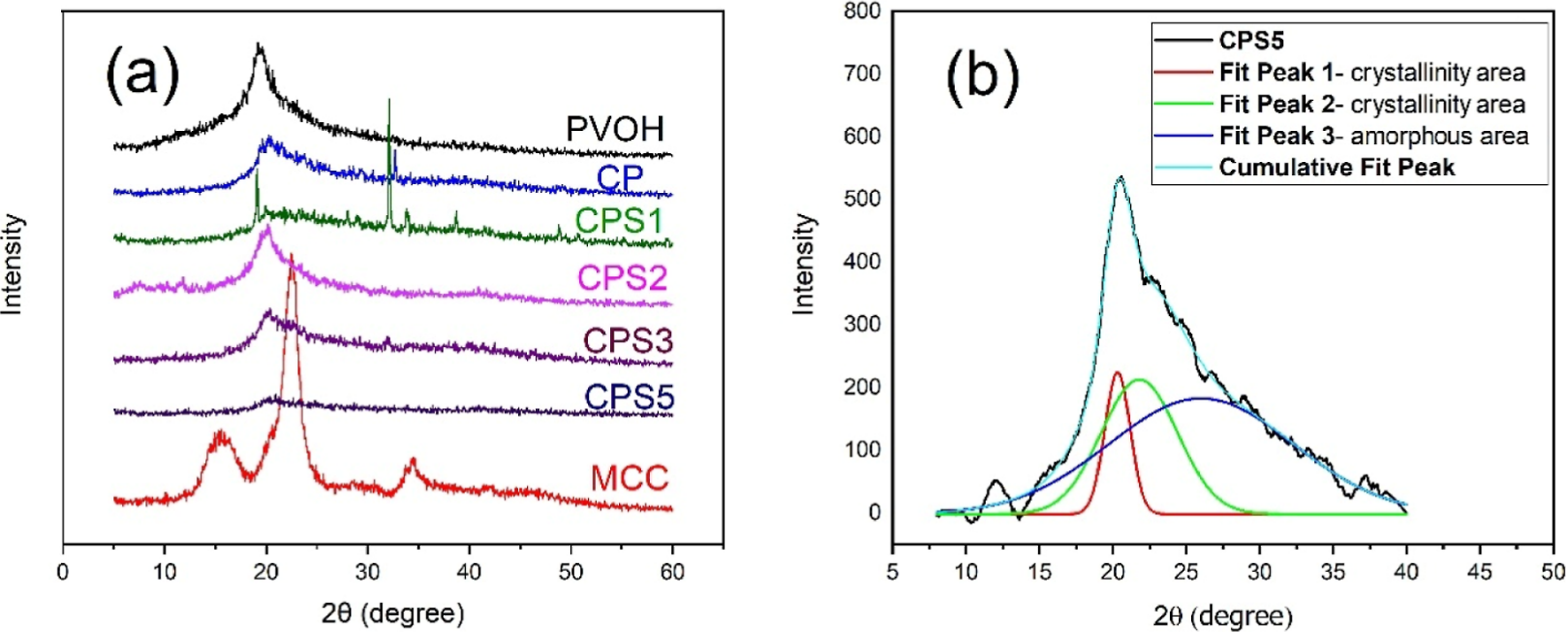
(a) XRD diffractograms of Microcrystalline cellulose (Cel), PVOH, CP,CPS1, CPS2, CPS3 and CPS5 blends. (b) Deconvolution CPS5 sample.

For the pure PVOH sample, the peak observed at 2θ = 19.3° is attributed to the 110 plane of an orthorhombic crystalline structure.^54^ In the CP, CPS1, CPS2, CPS3, and CPS5 blends, a broad peak was observed at 2θ = 19.5°, which may result from the overlap of two distinct peaks. The first corresponds to the orthorhombic reflection of PVOH, while the second indicates the transformation of cellulose from type I to type II allomorph, induced by the NaOH/urea solvent system.^55^ This peak exhibited greater broadening in blends with higher CS fractions due to the presence of CS, an amorphous polymer.^56,57^ Additionally, in the CPS1 blend two intense peaks are observed at 2θ = 19° and 2θ = 32°,^55^ corresponding, respectively, to the orthorhombic PVOH reflection and the cellulose type I → type II allomorphic transition promoted by the alkaline/urea system.

The crystalline index calculations, based on the deconvoluted areas in Figure 4b and the Supporting Information (Figure S4a to f **)**, revealed that the CP membrane exhibited a reduction in crystallinity compared to microcrystalline cellulose (MCC) (Table 4). This decrease can be attributed to solubilization, cross-linking, and regeneration processes, which disrupt the molecular ordering and lead to the formation of structures with lower crystallinity.^58^ Furthermore, this contributed to the reduction in crystalline. The interactions between cellulose/PVOH polymers with CS, an amorphous polymer, hinder the reorganization of cellulose–cellulose polymer chains during the regeneration process.^59^ Additionally, the cross-linking induced by ECH further disrupted the molecular arrangement, promoting a more disordered polymer structure and further decreasing crystallinity.^60^

**Table 4:** Crystallinity Index of the Pure Materials and the Produced Membranes

sample	% CI
MCC	82.02
PVOH	19.84
CP	43.07
CPS1	39.34
CPS2	33.78
CPS3	22.43
CPS5	10.20

### 3.4 TGA/DTG and DSC Analysis

The Figures S5a,b, S6a,b (Supporting Information), illustrates the thermal behavior of membranes with and without acetaminophen, as analyzed through TG/DTG. All samples exhibited a mass loss event between 50 and 100 °C in both TG/DTG and DSC curves which is attributed to water evaporation (Figure S7).

In the TG/DTG curves in Figures S5 and S6a,b, a second thermal event was observed in the Cel membrane and all polymer blends occurring between 230 and 275 °C. This event corresponds to the degradation of carbohydrates through the depolymerization of cellulose–cellulose chains, leading to glucose formation, as well as the decomposition of CS and acetaminophen.^61–64^ Additionally, the DTG curves revealed a mass loss between 400 and 500 °C in the CPS1, CPS2, CPS3, and CPS5 membranes, both with and without acetaminophen, which is associated with the thermal decomposition of PVOH.^65^

Thermogravimetric (TG) and derivative thermogravimetric (DTG) analyses enabled the determination of the initial degradation temperature (*T*_onset_), final degradation temperature (*T*_endset_), and maximum degradation temperature (*T*_max_), as presented in Table 5. The CP sample exhibited higher *T*_onset_ and *T*_max_ values compared to the Cel sample, indicating improved thermal stability. This enhancement is attributed to the formation of hydrogen bonding interactions between cellulose and PVOH chains, as corroborated by FTIR analysis. In contrast, a progressive decrease in *T*_onset_, *T*_endset_, and *T*_max_ was observed for the CPS1, CPS2, CPS3, and CPS5 samples. These results suggest that the incorporation of increasing amounts of chondroitin sulfate (CS) leads to a weakening of the hydrogen bonding network within the cellulose matrix, consistent with the FTIR findings.

**Table 5:** *T*_onset_, *T*_endset_, *T*_max_ and Residual Mass for all Samples

sample	*T*_onset_ (°C)	*T*_endset_ (°C)	*T*_max_ (°C)	residual mass at 600 °C (%)
Cel	162	370	246	41
CS	246	541	261	22
PVOH	247	479	364	22
CP	242	488	252	24
CPS1	188	327	256	30
CPS2	228	319	248	16
CPS3	215	304	237	22
CPS5	218	320	243	20
CPP	181	366	259	24
CPS1P	240	367	252	22
CPS2P	218	303	232	29
CPS3P	216	424	246	25
CPS5P	216	417	239	21

The polymer blends exhibited characteristic thermal events associated with all components in their formulation. However, as the CS content increased, both *T*_onset_ and *T*_max_ decreased relative to the CP membrane. This decline suggests that a higher CS concentration disrupted molecular organization, impeding the formation of a more crystalline structure during membrane fabrication, as corroborated by XRD analysis.

### 3.5 SEM Images

Figure 5 presents SEM topographic images of the surface and cryofractured regions of CP, CPS1, CPS2, CPS3, and CPS5 membranes incorporated with acetaminophen.

**Figure 5:**
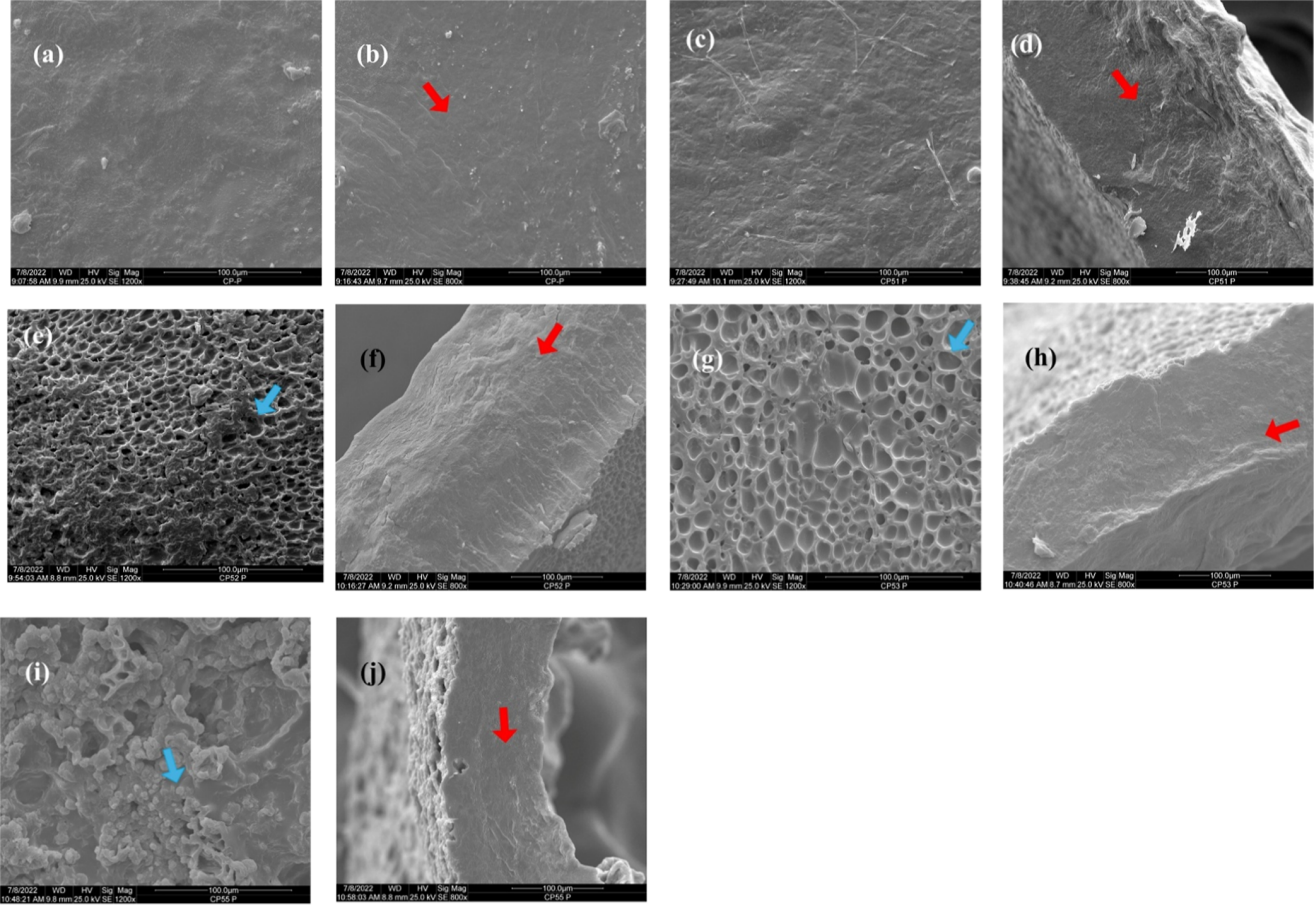
SEM images of the membrane surface magnified 1200 times (a, c, e, g and i). SEM images magnified 800 times in the fractured regions (b, d, f, h and j). Red arrows indicate compact regions without pores. Blue arrows indicate pore formation on the surface. (a) and (b): CP. (c) and (d): CPS1. (e) and (f): CPS2. (g) and (h): CPS3. (i) and (j): CPS5.

As the chondroitin sulfate content increases, a distinct roughness pattern emerges, as observed in the SEM images of CPS2, CPS3, and CPS5. This phenomenon is attributed to the increased presence of –SO_3_^–^ groups, which enhance electrostatic repulsion between the polar groups of the polymer chains, as showed in Figure 1d, thereby promoting pore formation.^66^ In the CPS5 membrane, where the chondroitin sulfate concentration is highest, spherical structures were observed on the surface. This effect is consistent with the findings of Guo,^67^ who developed PVOH/chondroitin sulfate blends via electrospinning and reported that higher chondroitin sulfate content led to the formation of spherical structures due to sulfate group repulsion. Additionally, the formation of surface pores is attributed to the coagulation process induced by 5% (v/v) H_2_SO_4_, indicating successful regeneration of the cellulose matrix.^65–70^ According to Yahya,^71^ the presence of surface roughness and porosity may enhance the performance of membranes by facilitating cell adhesion. Scanning electron microscopy (SEM) analysis of the cryofractured cross sections revealed a compact, homogeneous, and smooth internal morphology, indicating that porosity is predominantly restricted to the surface layer. These findings are consistent with the results of Jiang, Hongo and Grazioli,^72–74^ who observed similar morphological characteristics in regenerated cellulose membranes.

### 3.6 Physicochemical Analysis

The results presented in Table 6 indicated a significant reduction in the MC, FHC, and WVP values in the CP samples compared to the Cel sample. This reduction is attributed to the decreased availability of hydroxyl groups capable of interacting with water molecules, resulting from the cross-linking reaction with epichlorohydrin, as indirectly supported by the FTIR results and the water-interaction behavior of the membranes. Furthermore, the increased structural compactness observed in the CP sample relative to the Cel membrane evident in the SEM images (Figure 6) may have further contributed to the reduction in these physicochemical parameters. The FHC is directly related to wound dressing’s ability to absorb, retain, and manage exudate over time.^75^ All samples demonstrated high FHC values, as presented in Table 5. The Cel membrane exhibited an FHC of 9.41 g·cm^–2^. The FHC of the PVOH membrane could not be determined due to the high solubility of the polymer in water, which compromised the structural integrity of the sample during testing.

**Table 6:** Moisture Content Data (% MC); Fluid Handling Capacity (FHC); Water Vapor Permeability (WVP) of Membranes^a^

sample	MC (%)	FHC (g 10 cm^–2^ 24 h^–1^)	WVP (10^–6^) (g Pa^–1^ s^–1^ m^–1^)
Cel	22.15^*a*^	9.41^*a*^	5.73^*a*^
PVOH	-	-	2.71^*cd*^
CP	10^*b*^	3.63^*b*^	4.41^*ab*^
CPS1	13.12^*b*^	3.48^*b*^	3.09^*bc*^
CPS2	10.99^*b*^	3.47^*b*^	2.01^*cde*^
CPS3	9.74^*b*^	4.26^*b*^	1.24^*de*^
CPS5	10.31^*b*^	5.15^*b*^	0.78^*e*^

a *Different letters in the same column (*a*–*d*) indicate significant differences (*P* < 0.05) according to the Tukey test.

**Figure 6:**
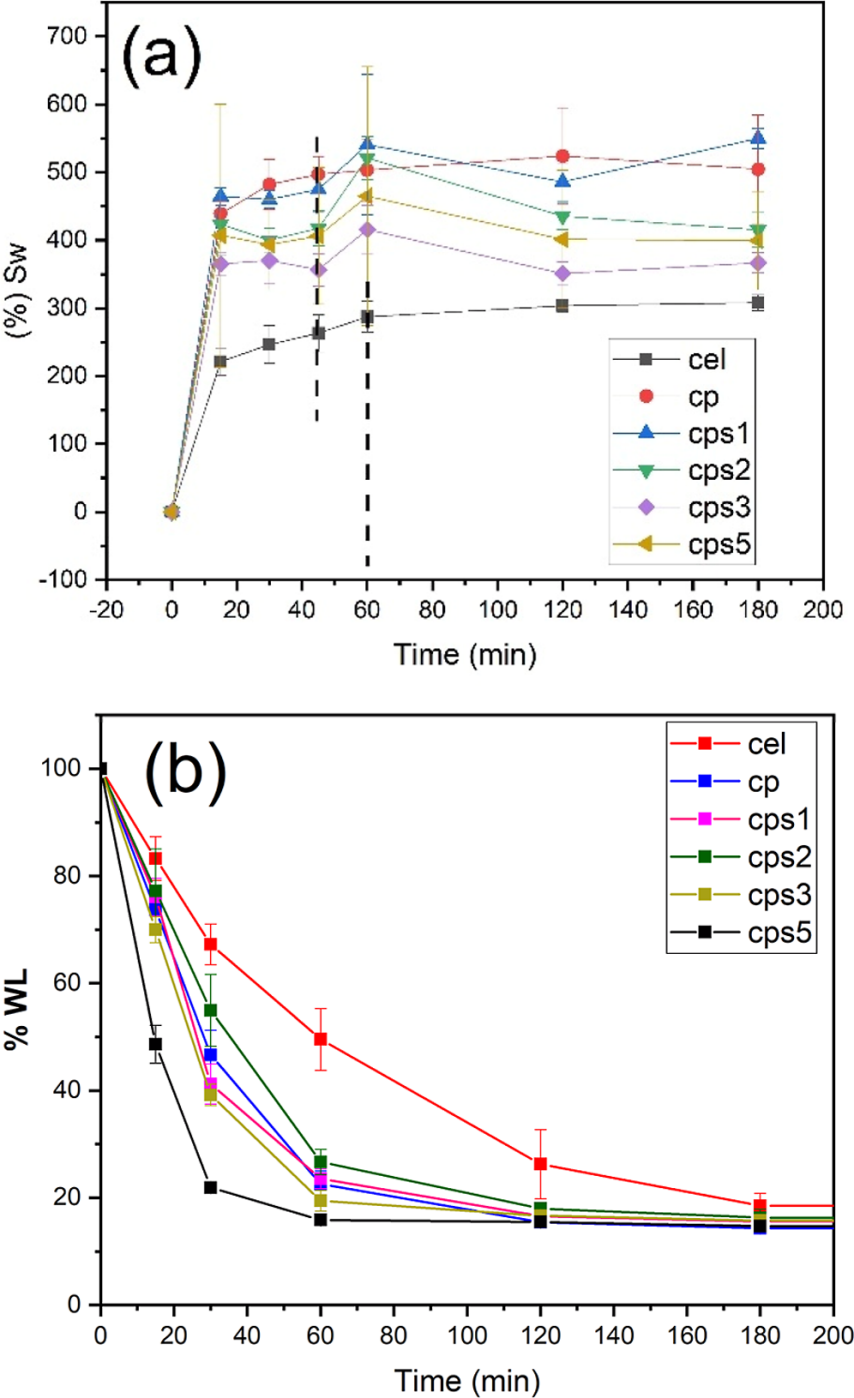
Water absorption and loss kinetics. (a) Swelling behavior of the membranes. (b) Water loss from the membranes.

Following the incorporation of CS, a reduction in FHC was observed in comparison to the pure cellulose membrane (Cel), with values ranging from 3.4 to 5.5 g 10 cm^–2^ 24 h^–1^. The increasing concentration of CS did not cause significant changes in the moisture content MC and fluid FHC values.

According to Kimura,^76^ wound exudate production may vary between 3.4 and 5.1 g per 10 cm^2^ over a 24 h period. Despite the observed decrease in FHC, the membranes containing CS still demonstrate appropriate absorptive capacity for use as wound dressings, as they remain within the expected exudate absorption range.

The reduction in WVP observed for the membranes CP, CPS1, CPS2, CPS3, and CPS5 may represent a beneficial characteristic, as lower WVP values are associated with reduced water loss by evaporation.^77^ Maintaining adequate moisture levels is essential to create an optimal environment for wound healing.^78^ Injured skin experiences transepidermal water loss at a rate approximately 20 times higher than that of intact skin. Moreover, the healing process is generally accelerated in moist environments compared to dry conditions.^79^

Figure 6 presents the swelling kinetics and water loss profiles of the membranes. As illustrated in Figure 6a, the pure cellulose membrane required 60 min to reach swelling equilibrium, whereas the membranes (CP, CPS1, CPS2, CPS3, and CPS5) achieved equilibrium within 40 min. Membranes incorporating chondroitin sulfate exhibited significantly enhanced swelling capacity compared to the pure cellulose membrane. This observation agrees with previous findings by Oprea and Vlad-Bubulac,^12,13,66^ where the ionization of carboxyl (–COOH) and sulfate (–CH_2_SO_3_H) groups to their anionic forms (–COO^–^ and –CH_2_SO_3_^–^) induced electrostatic repulsion between polymer chains, thereby increasing water absorption capacity and swelling behavior.

The CPS2, CPS3, and CPS5 membranes exhibited significantly reduced swelling capacity compared to the CP and CPS1 membranes. This behavior can be attributed to the decreased concentrations of cellulose and poly­(vinyl alcohol) (PVOH) while maintaining constant ECH content, which promotes the formation of a more densely cross-linked polymer network. The increased cross-linking density restricts chain mobility, leading to a more compact structure with consequently lower swelling capacity, as described by the Flory–Rehner theory of polymer network.^76^ These findings are consistent with previous reports indicating that increased cross-linking density reduces the free volume within the polymer matrix, thereby limiting water absorption.^77^ This structural effect is further evidenced in the water loss profiles (Figure 6b), where highly cross-linked membranes exhibited faster water evaporation rates. The rapid water loss suggests surface-dominated evaporation processes in these membranes, as opposed to bulk diffusion-controlled mechanisms observed in less cross-linked systems.

To facilitate the interpretation of the experimental results, Table 7 summarizes the main observations obtained from the different characterization techniques and their corresponding structural implications.

**Table 7:** Summary of Structural, Morphological, and Physicochemical Characteristics of the Membranes

technique	main observation	structural implication
FTIR	broad band at 3260–3300 cm^–1^	hydrogen-bonded hydroxyl groups from cellulose and PVOH
FTIR	band near 1235 cm^–1^	sulfate groups from chondroitin sulfate
FTIR	increase in the C–O–C region (≈1085–1020 cm^–1^)	formation of ether linkages due to epichlorohydrin cross-linking
FTIR deconvolution	changes in OH stretching region	modification of hydrogen bonding network
XRD	diffraction pattern characteristic of cellulose II	regeneration of cellulose after dissolution in NaOH/urea
SEM	continuous membrane structure with morphology influenced by CS content	structural organization of the polymer network
SEM	changes in surface morphology with increasing CS concentration	influence of CS incorporation on membrane microstructure
water interaction tests	variation in MC, FHC and WVP	hydrophilicity influenced by CS incorporation and cross-linking

### 3.7 Acetaminophen Release Kinetics

The acetaminophen release profiles as a function of time, presented in Figure S8, reveal two phases in the process during the release. An initial rapid release phase, commonly referred to as the “burst effect,” occurs within the first 2 h, followed by a sustained and slower release phase. The burst release is likely attributed to the diffusion of acetaminophen molecules located near or on the surface of the membrane. In contrast, the subsequent slower release phase is predominantly governed by the diffusion of the drug through the polymer matrix, involving interactions between polymer chains that hinder the mobility of the encapsulated acetaminophen.^80,81^

Two distinct groups were identified based on the acetaminophen release profiles, Figure 7. The first group, comprising the Cel, CP, and CPS1 samples, exhibited higher drug release, whereas the second group, including CPS2, CPS3, and CPS5, demonstrated lower acetaminophen release. A similar trend was reported by Oprea.^12,13^

**Figure 7:**
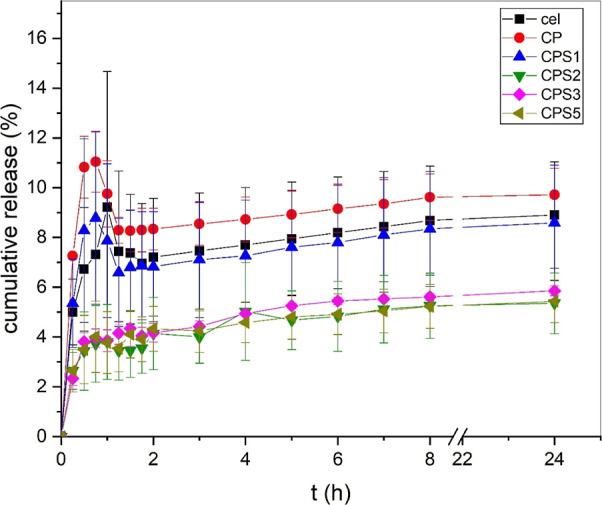
In vitro diffusion of acetaminophen through a Franz cell in phosphate buffer (pH 7.4). Acetaminophen release expressed as % (mass per area) over time (h).

Although increased porosity is generally expected to improve drug release by providing more diffusion pathways, as occurs with sample CPS1, the significantly reduced release rate for samples CPS2–CPS5 suggests that other factors predominate at higher CS concentrations. The alteration of pore structures on the membrane surfaces, shown in SEM images (Figure 5), may have caused the formation of smaller pathways or tortuosity, resulting in a reduced drug release rate. Furthermore, the higher concentration of CS may have increased the hydrophilicity of the membranes, leading to their swelling and blockage of the surface pores. Additionally, the increased concentration of chondroitin sulfate may lead to a more compact or cross-linked polymeric network, thus hindering internal drug diffusion.

The kinetics of acetaminophen release from all membrane samples were evaluated by plotting the ratio of the cumulative drug mass released at each time point (*M*_*t*_) to the equilibrium drug mass (*M*_e_) as a function of time. The kinetic parameters for each model were determined by fitting the experimental data to the respective mathematical equations outlined in Table 8. All curve-fitting and analyses were performed using MATLAB R2016b. For the first-order, Peppas and Sahlin, and Ritger-Peppas models,^82,83^ only the release data corresponding to up to 60% of the total release profile were considered, in accordance with model assumptions. For the Schott model,^82^ the entire data set from 0 to 24 h was employed in the analysis.

**Table 8:** Kinetic Models for Swelling^a^

model	equation	refs
Korsmeyer Peppas	$F=\frac{M_{t}}{M_{e}}=kt^{n}$	^83^
first order	$\ln\left(W_{e}-W_{t}\;\right)=\ln W_{e}-kt$	^85^
second order	$\frac{t}{W_{t}}=B+A\times t$	^84^
Peppas Sahlin	$F=\frac{M_{t}}{M_{e}}=k_{1}t^{n}+k_{2}t^{2n}$	^82^

a * *M*_*t*_ = mass of water absorbed at time *t*; *M*_e_ = mass of water at equilibrium; *t* = time (min); In the Korsmeyer and Peppas (1987) model, *k* is the kinetic constant and *n* is the diffusion constant. In the Schott (1992) model, *W*_*t*_ is the swelling at time *t*, *B* = 1/*k*_s_. *W*_e_^2^ and *A* = 1/W_e_, where *W*_e_ is the theoretical swelling at equilibrium and *k*_s_ is the swelling constant. In the Peppas and Sahlin (1989) model, *k*_1_ is the kinetic constant of the diffusion contribution; *k*_2_ is the kinetic constant of the relaxation contribution and n is the diffusion constant.

Based on the values of the diffusional exponent (*n*) and the kinetic constants (*k*_1_ and *k*_2_) obtained from the Peppas and Sahlin^82^ model, the relative contribution of polymer chain relaxation (*R*) to the Fickian diffusion component (*F*) was calculated for all membranes using eq 11.

11 $$\frac{R}{F}=\frac{k_{2}}{k_{1}}t^{n}$$

After 24 h of drug release analysis, distinct release profiles were observed between the membrane groups. The cellulose (Cel), CP, and CPS1 samples demonstrated a cumulative acetaminophen release (*M*_e_) of approximately 8–10%, while the CPS2, CPS3, and CPS5 samples showed significantly lower release (*M*_e_ ≈ 5%). This 2-fold difference in drug release correlates with the cross-linking density variations among the membranes, where higher cross-linking (CPS2-5) restricts polymer chain mobility and reduces drug diffusion rates. Based on the linear regression coefficients (*R* ^2^ > 0.95) presented in Table 9, it can be concluded that the release kinetics of acetaminophen are well described by the models proposed by Ritger and Peppas,^83^ Peppas and Sahlin^82^ and Schott,^84^ with the latter providing the best fit to the experimental data (*R* ^2^ > 0.99) and theoretical amount of acetaminophen released close to those obtained experimentally. In the Ritger and Peppas^82^ models, the diffusional exponent *n* characterizes the release mechanism: for *n* < 0.5, the release is classified as quasi-Fickian; for 0.5 < *n* < 1, the release follows Fickian diffusion; and for *n* > 1, the mechanism is considered anomalous or non-Fickian. The constants *k*, *k*_1_, and *k*_2_ represent the release rate constants associated with the respective kinetic models.

**Table 9:** Kinetic Parameters of Acetaminophen Release from the Adjustments of the Kinetic Models for the Membrane’s Cel, CP, CPS1, CPS2, CPS3 e CPS5

	parameters	Cel	CP	CPS1	CPS2	CPS3	CPS5
Korsmeyer-Peppas	*k*	0.7753	0.8525	0.6613	0.683	0.6800	0.6912
	*n*	0.1304	0.2026	0.259	0.1200	0.2814	0.1772
	RMSE	0.0590	0.0730	0.1104	0.0542	0.0587	0.04523
	*R* ^2^	0.9578	0.9525	0.9319	0.9525	0.9518	0.9512
Peppas-Sahlin	*k*_1_	0.7753	0.8569	1.019	0.682	0.6896	0.6912
	*k*_2_	1.99 × 10^–10^	1.46 × 10^–11^	1.30 × 10^–11^	1.60 × 10^–11^	2.80 × 10^–13^	1.79 × 10^–10^
	*n*	0.1304	0.2026	0.2585	0.1280	0.2814	0.1772
	RMSE	0.0590	0.0730	0.1104	0.0542	0.0587	0.0452
	*R* ^2^	0.9578	0.9525	0.9319	0.9525	0.9518	0.9512
	*k*_Schott_	5.944	9.102	5.924	5.886	5.262	6.186
Schott	*A*	1.967	1.810	2.040	3.226	2.952	3.190
	B	0.650	0.350	0.703	1.768	1.656	1.650
	theortical release (mg cm^–2^)	0.500	0.550	0.480	0.300	0.330	0.310
	experimental release (mg cm^–2^)	0.480	0.540	0.470	0.290	0.320	0.290
	*R* ^2^	0.998	0.998	0.997	0.997	0.998	0.998
first order	*k*_3_	–8.294	–3.069	–16.55	–3.479	–3.73	–5.156
	ln *W*_*e*_	7.394	6.422	8.178	4.372	4.933	4.829
	*R* ^2^	0.8374	0.5687	0.9435	0.66	0.673	0.80

According to the Schott model, the theoretical maximum concentration of acetaminophen decreased with the increasing concentration of chondroitin sulfate (CS) in the membrane composition. The comparison between the Cel and CP membranes revealed an increase in the rate constant, indicating a faster drug release. In contrast, the incorporation of CS led to a progressive reduction in the release rate for the CPS1, CPS2, and CPS3 membranes.

Interestingly, for the CPS5 membrane, a reversal of this trend was observed, with an increase in the rate constant, suggesting a distinct drug release behavior at higher CS concentrations. The results presented in Table 9 indicate that the values of the exponential constant (*n*) were below 0.5 for both the Ritger and Peppas,^82^ characterizing a quasi-Fickian diffusion mechanism. However, it is important to note that several authors have classified diffusion as Fickian even when *n* < 0.5 in cellulosic systems. For instance, Dai and Huang and Xia^86,87^ reported Fickian diffusion behavior in hydrogels composed of carboxymethyl cellulose and microcrystalline cellulose, respectively. The *n* values obtained in the present study suggest that chain relaxation exerts a minimal influence on the drug release kinetics, as also supported by Unagolla and Jayasuriya^88^.

The ratio between the contributions of polymer chain relaxation (*R*) and diffusion (*F*) was evaluated as a function of time. For all membrane formulations and throughout the analyzed time intervals, the *R*/*F* values remained below 1. According to Unagolla and Jayasuriya,^88^ an *R*/*F* value equal to 1 indicates that the release mechanism is equally governed by diffusion and polymer chain relaxation. Values greater than 1 indicate a predominance of relaxation-controlled release, whereas values lower than 1 suggest that the release process is mainly governed by diffusion (see Figure S8a–d).

The data presented agree with the kinetic modeling results summarized in Table 9, further reinforcing the conclusion that the diffusion process plays a dominant role in acetaminophen transport through these cellulose-based membranes. This is consistent with the behavior typically observed in semicrystalline cellulose matrices, where chain mobility is restricted, thereby limiting the influence of polymer relaxation.

Such findings are significant, especially considering the potential application of these membranes in transdermal drug delivery, where controlled release kinetics are essential.

## 4 Conclusions

Ternary polymeric membranes were successfully fabricated through the cross-linking of cellulose (Cel), poly­(vinyl alcohol) (PVOH), and chondroitin sulfate (CS) using ECH as the cross-linking agent. The presence of CS led to a reduction in FHC, MC, and WVP compared to the pristine cellulose membrane. These changes resulted in membranes with physicochemical properties favorable for transdermal delivery applications. Kinetic analysis demonstrated that all membrane formulations exhibited drug release profiles consistent with Fickian diffusion mechanisms (*n* < 0.45 in Korsmeyer-Peppas modeling).

FTIR analysis and physicochemical characterization revealed a direct correlation between membrane composition and both (i) polymer network architecture and (ii) drug release kinetics. Two distinct release profiles were observed: membranes with lower CS concentration (Cel, CP, CPS1) released approximately 10% of acetaminophen within 24 h, while those with higher CS concentration (CPS2–CPS5) exhibited a significantly lower release (∼5%, *p* < 0.05), indicating a controlled and sustained release pattern. However, it would be beneficial expand on how membrane composition (e.g., chondroitin sulfate content) might modulate the *R*/*F* ratio across different formulations.

All membrane formulations displayed low crystallinity and surface porosity, characteristics which enhance their suitability for wound healing applications. These findings emphasize the critical role of biopolymer composition and network architecture in tuning membrane performance. Moreover, the fabrication method proposed herein offers a simple, scalable, and cost-effective strategy for producing biopolymer-based transdermal membranes and wound dressings with controlled drug delivery functionality.

## Supplementary Material



## Notes

FTIR spectra of raw materials; FTIR spectral deconvolution data; thermal analysis curves (TG/DTG and DSC) of raw material and blends; ratio between the contributions of polymer chain relaxation (*R*) and diffusion (*F*) as function of time for all membrane formulations (PDF)
