# Supplementary material for: Influence of Chondroitin Sulfate Concentration on the Physicochemical Properties of Acetaminophen-Releasing Cellulose/PVOH Membranes
Source: ACS Omega. 2026 Apr 16;11(16):24650–64. doi: 10.1021/acsomega.6c01092 (PMC13130104; doi:10.1021/acsomega.6c01092)
Supplement: Supplementary file 1 [file ao6c01092_si_001.pdf]

Influence of Chondroitin Sulfate Concentration on the Physicochemical Properties of Acetaminophen-Releasing Cellulose/PVOH Membranes

Correspondence: Juliano Brisola (julianobrisola@gmail.com) / **Gizilene M. de Carvalho** (gizilene@uel.br)

Juliano Brisola[a,*], Paula Paulino Silva[a], Francisnara Tonholi[a], Jéssica Bassetto Carra[a] Rúbia Casagrande[b], Paulo Rodrigo Stival Bittencourt[c], Gizilene Maria de Carvalho[a,*]

[a] *Department of Chemistry, State University of Londrina, Londrina, PR, Brazil*

[b] *Department of Pharmaceutical Sciences, State University of Londrina, Londrina, PR, Brazil*

[c] *Department of Chemistry, Federal University of Technology – Paraná (UTFPR), Medianeira Campus, Medianeira, Paraná, Brazil*

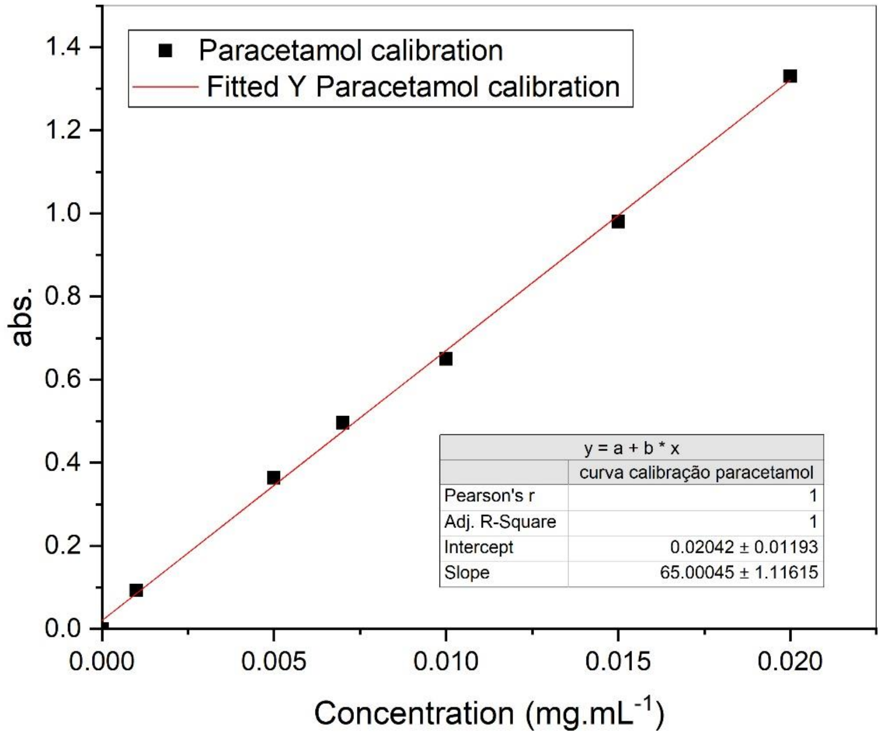

Fig. S1. Paracetamol Calibration

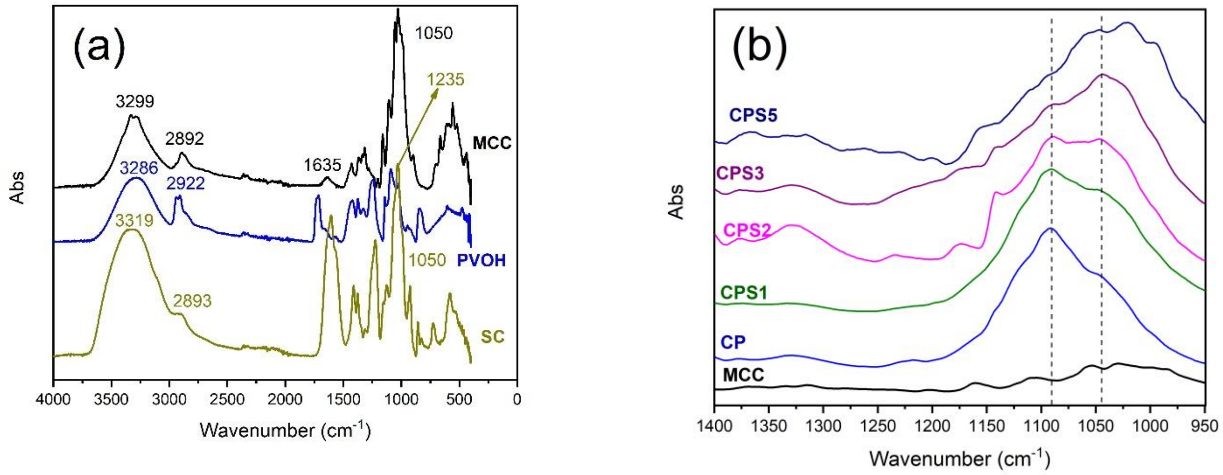

Fig. S2. **(a)** Spectra of the neat components: cellulose, PVOH, and chondroitin sulfate (CS). **(b)** Spectral detail in the region between 1400 and 950 cm^{-1}, highlighting characteristic vibrational modes.

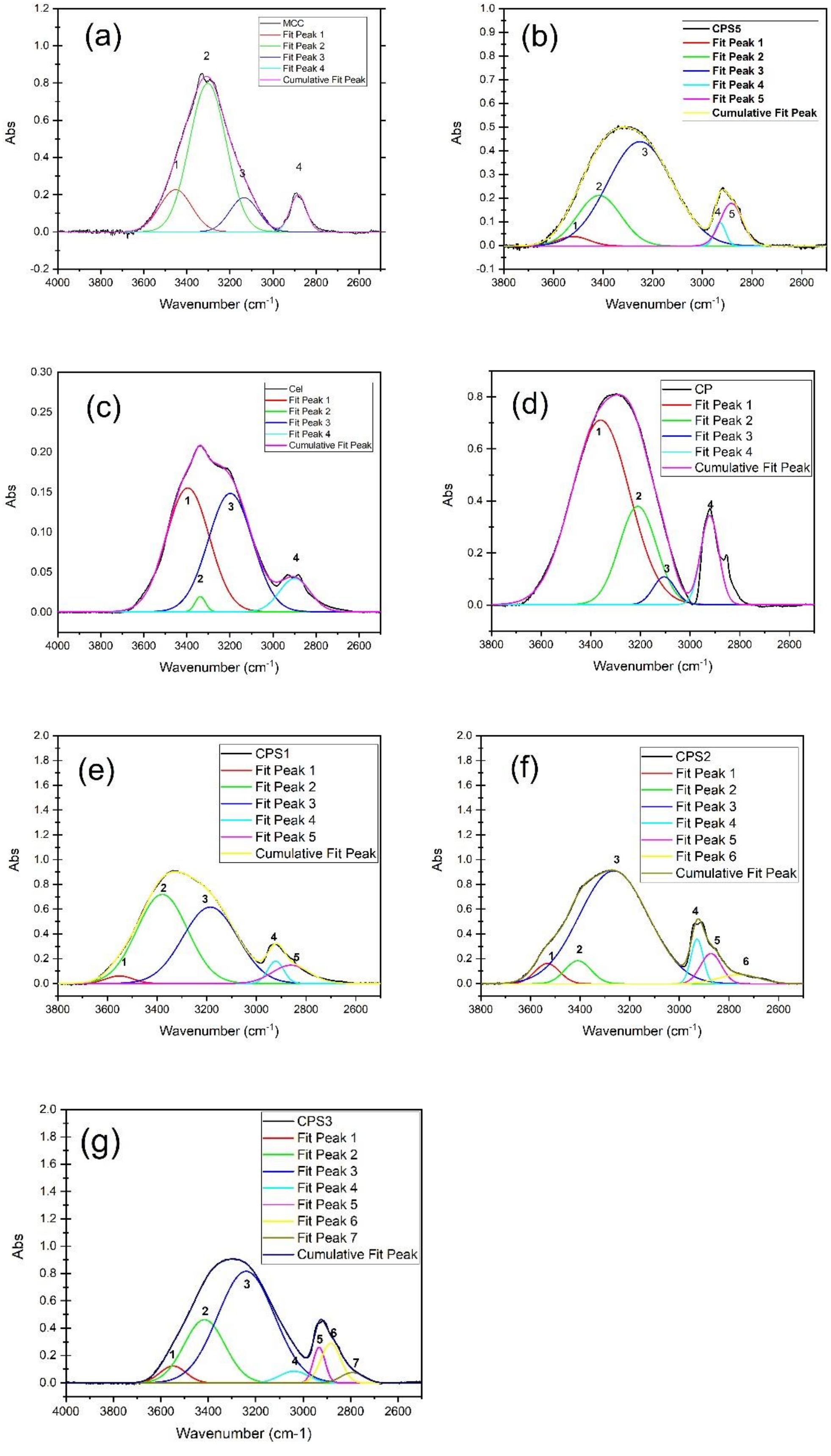

Fig. S.3 FT-IR Deconvolution Results in the 3500 to 2500 cm^{-1} Region of the Produced Membranes. **(a)**. MCC. **(b)**. CPS5. **(c)**. Cel. **(d)**. CP. **(e)**. CPS1. **(f)**. CPS2. **(g)**. CPS3

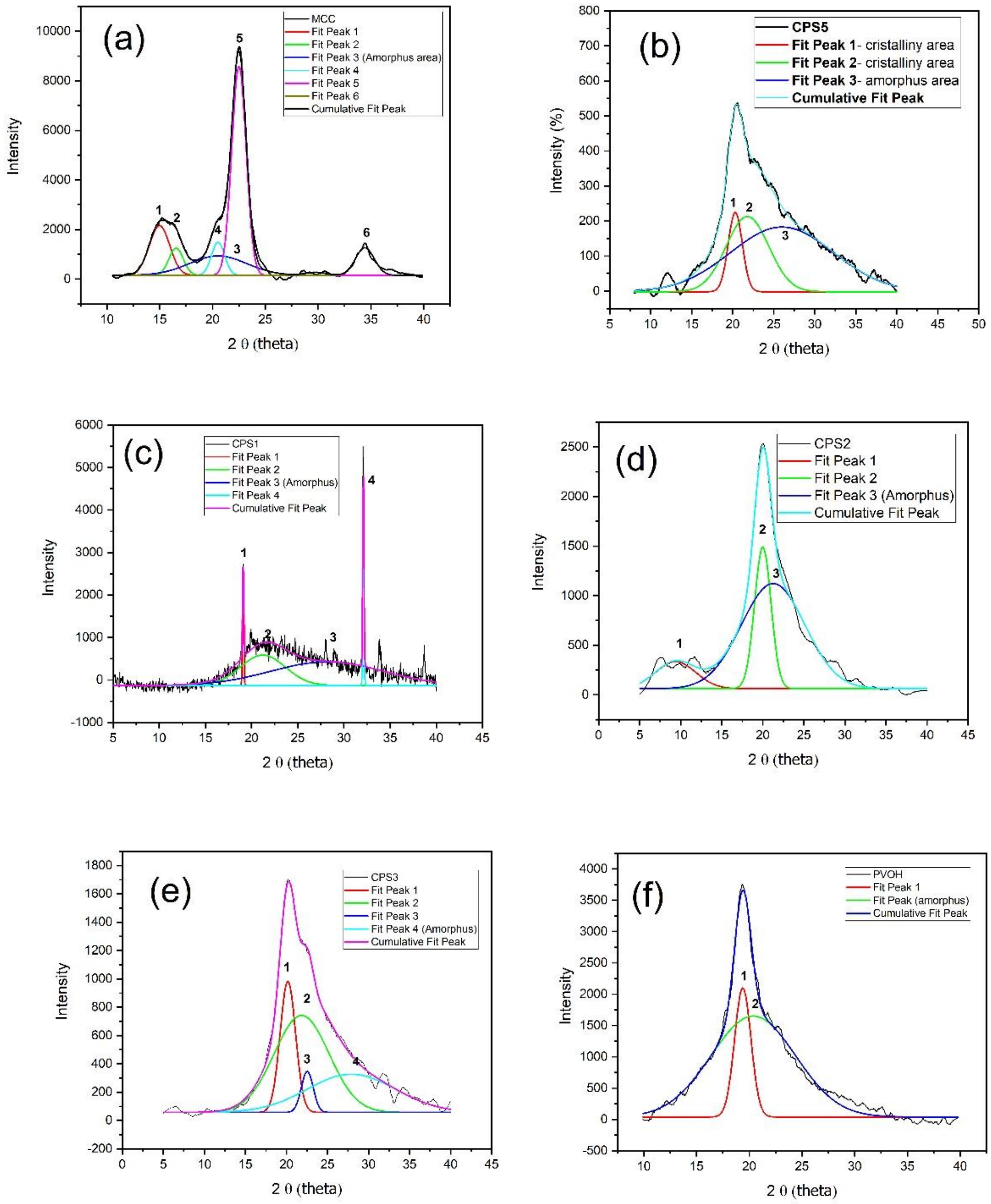

Fig. S.4. Deconvolution Results of the Diffractograms of All Membranes. (**a**). MCC. (**b**). CPS5. (**c**). CPS1. (**d**). CPS2. (**e**). CPS3. (**f**). PVOH

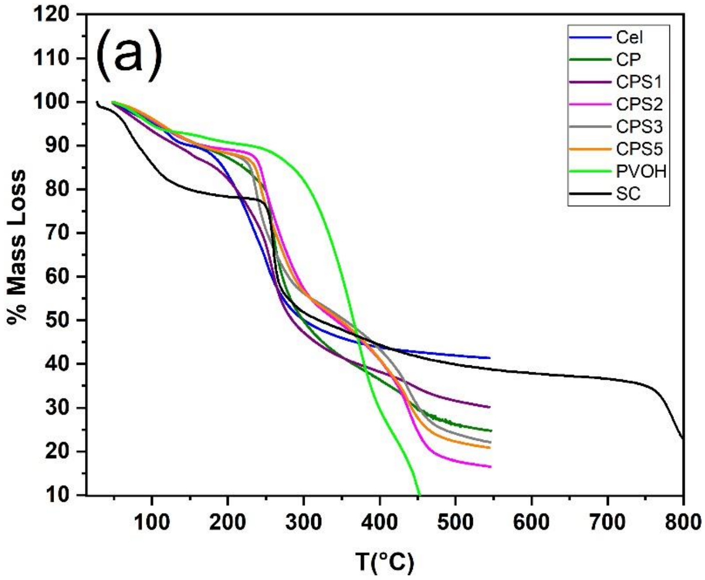

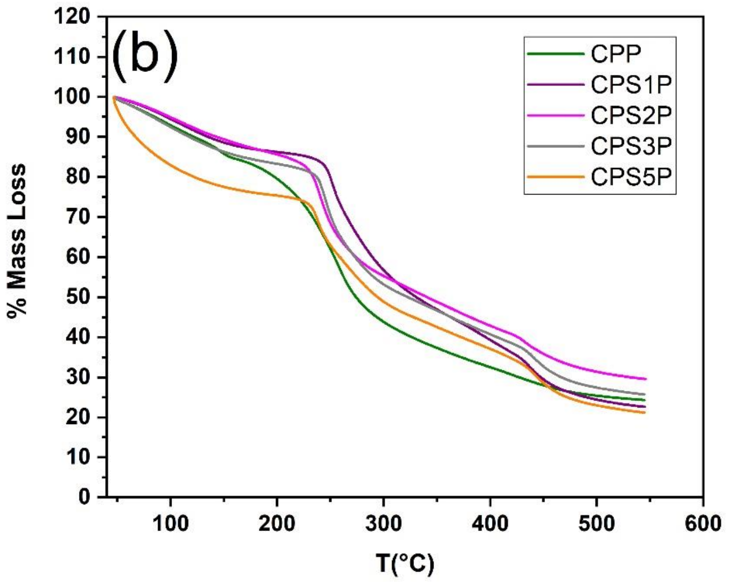

Fig. S5. **(a)** TG curves of the samples: Cel, PVOH, SC, and the membranes CP, CPS1, CPS2, CPS3, and CPS5 without paracetamol. **(b)** TG curves of the membranes CPP, CPS1P, CPS2P, CPS3P, and CPS5P after paracetamol incorporation.

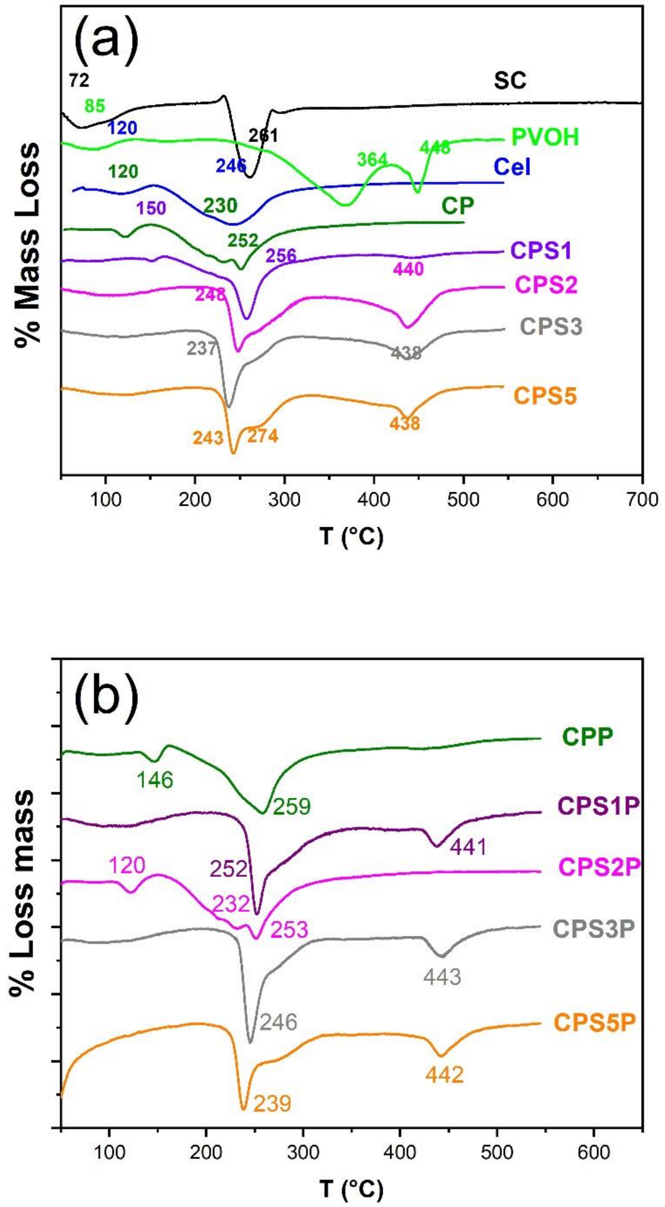

Fig. S6. **(a)** DTG curves of the samples: Cel, PVOH, SC, and the membranes CP, CPS1, CPS2, CPS3, and CPS5 without paracetamol. **(b)** DTG curves of the samples: Cel, PVOH, SC, and the membranes CPP, CPS1P, CPS2P, CPS3P, and CPS5P after paracetamol incorporation.

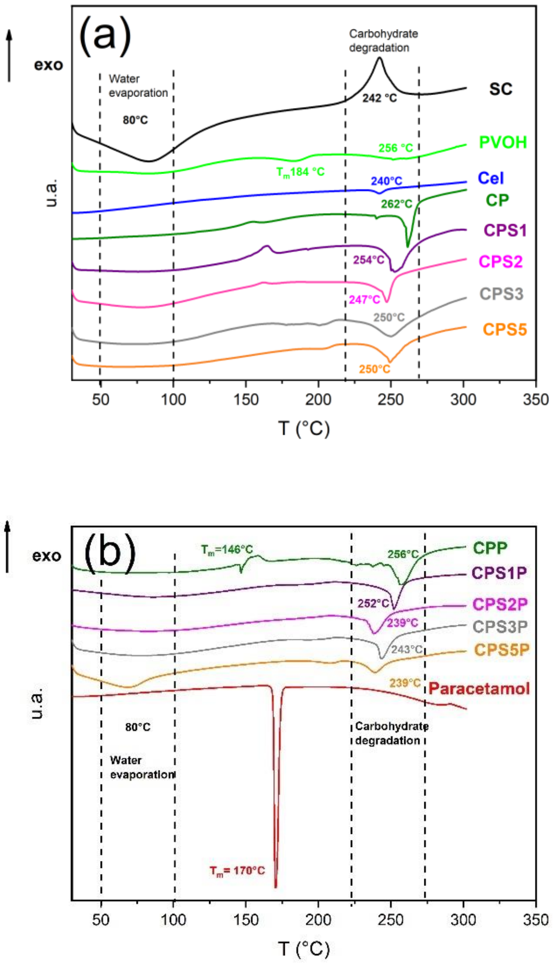

Fig. S7 (a) DSC thermograms of the samples: Cel, PVOH, SC, and the membranes CP, CPS1, CPS2, CPS3, and CPS5 without paracetamol. **(b)** DSC thermograms of pure paracetamol and the membranes CPP, CPS1P, CPS2P, CPS3P, and CPS5P after paracetamol incorporation.

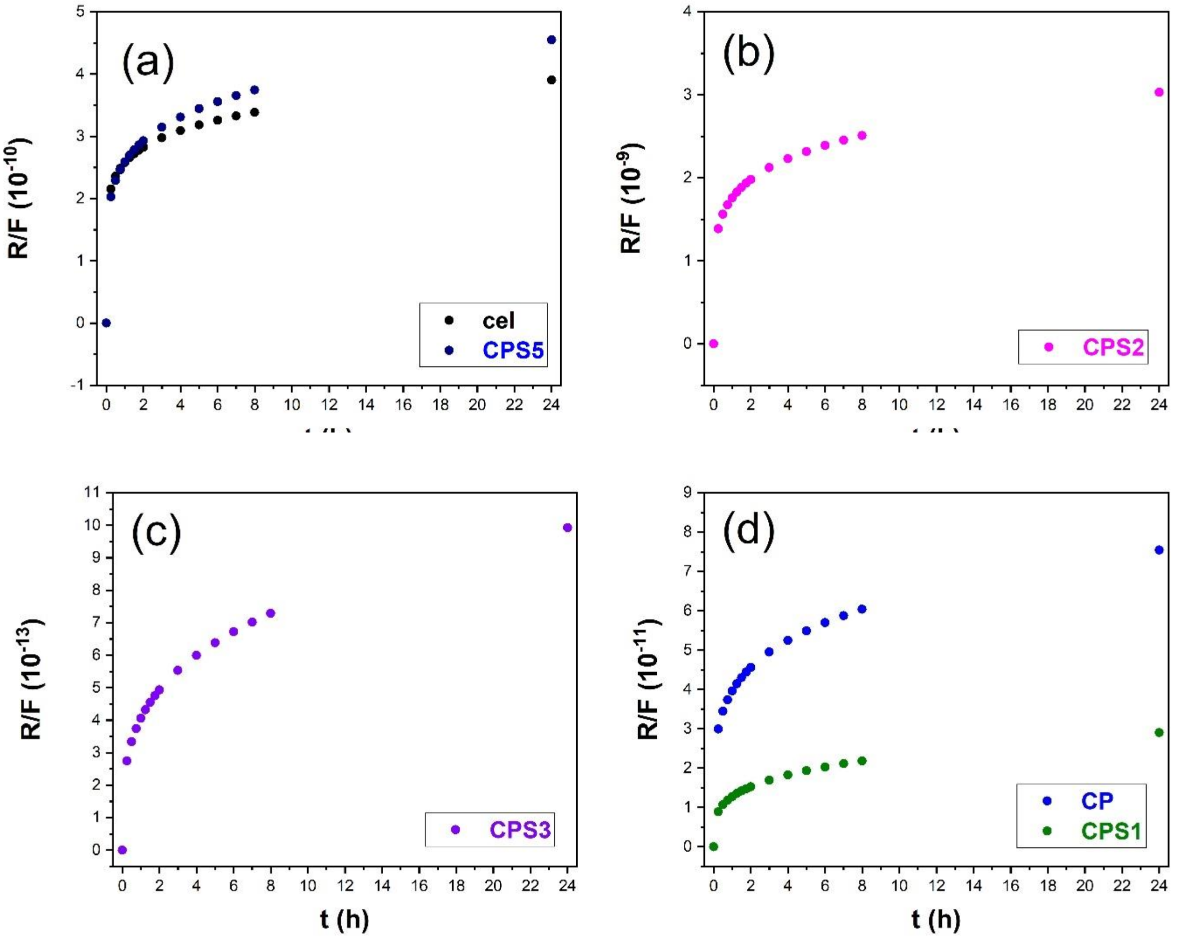

Fig S8. Ratio of relaxation contribution/Fickian contribution (R/F) with respect to time. **(a)** cel e CPS5. **(b)** CPS2. **(c)** CPS3. **(d)** CP e CPS1.
